# Evaluation of Knowledge and Competencies in Sexual and Reproductive Health Care Using an Escape Room with Scenario Simulations

**DOI:** 10.3390/nursrep14020052

**Published:** 2024-03-22

**Authors:** Juan Miguel Martínez-Galiano, Manuel Gonzalez-Cabrera, Julian Rodriguez-Almagro, Antonio Hernández-Martínez

**Affiliations:** 1 Department of Nursing, University of Jaen, 23071 Jaén, Spain; mgonzale@ujaen.es; 2 Consortium for Biomedical Research in Epidemiology and Public Health (CIBERESP), 28029 Madrid, Spain; 3 Department of Nursing, Physiotherapy and Occupational Therapy, University of Castilla-La Mancha, 13071 Ciudad Real, Spain; julianj.rodriguez@uclm.es (J.R.-A.); antonio.hmartinez@uclm.es (A.H.-M.)

**Keywords:** escape room, gamification, simulation, nursing, sexual and reproductive health care, OSCE

## Abstract

To determine the usefulness of combining two methodologies (OSCE and escape room) in a scenario simulation to evaluate a subject, and determine the evaluation of the students of this experience. An observational cross-sectional study was carried out with students enrolled in a sexual and reproductive health-care course as a part of their nursing degree. The students had to solve four clinical cases based on the contents of the teaching practices of the subject by solving clues that led them to carry out procedures and techniques and provide care in scenario simulators. Students evaluated the experience using the GAMEX (Gameful Experience in Gamification) scale. Mean differences were estimated with their respective 95% confidence intervals. A total of 124 students participated. Of these, 63.7% (79) solved the clinical cases with their knowledge and skills. Most (80.6%, 100) students stated that they completely remembered and applied the knowledge of the topic during the game. Almost all (98.4%, 122) would recommend this experience. The dimensions with the best rating on the GAMEX scale were “fun”, with an average score of 4.7 points (0.49), followed by “critical thinking”, with 4.2 (0.59). Women presented statistically better scores than men (mean difference: 1.58; 95% CI: 0.55, 2.61). The OSCE combined with an escape room using scenario simulations may be a useful tool to evaluate the subject. In addition, the students were satisfied, had fun, and recommended the experience. This study was not registered.

## 1. Introduction

The traditional teaching model centers on lectures in which the teacher has the greatest role and the student a secondary role. This teaching model has been shown not to be the most effective pedagogical technique for teaching disciplines such as nursing in the current social context [1,2]. In the teaching–learning process of nursing, theoretical and procedural content and the development of skills and abilities must be dynamically integrated, resulting in a complex process that challenges teachers and students [3].

Teaching methodology that promotes active learning combined with ICT (information and communication technology) in which student participation is predominant is being implemented in university education with good results [4,5,6,7]. Within this active methodology, gamification plays a prominent role. Gamification consists of using game elements, or the games themselves, in contexts other than a game, and in this case, within education to improve academic performance [8]. For example, different games such as puzzles, role-playing games, or crossword puzzles, among others, have been proven effective in the learning process [9] and students show interest in them. Despite this, active learning strategies are often not implemented in the academic institutions of higher education where nursing is taught. This is due, among other reasons, to the fact that while those who carry out their work in the classrooms have comprehensive training in their academic field, they may not have the same level of didactic–pedagogical training [10].

The escape room is another didactic technique that falls within gamification [11], and consists of a collaborative live-action game that requires participants to solve clues to escape from a closed room [12]. Its use as a pedagogical technique in teaching has experienced a great boom in recent times [13], and has been carried out in higher education in different health science disciplines [14,15]. In addition, it has been well received by the students, and the results obtained have been good [16,17,18]. Different escape room experiences have been carried out in countries such as the USA or Spain in the teaching of nursing and for different subjects, such as anatomy [19], cardiovascular nursing [20], and community nursing [21].

The objective structured clinical evaluation (OSCE) test comprises several stations in a circuit through which all the students rotate. It is designed to assess student performance in specific clinical situations, where their knowledge, clinical reasoning, skills, abilities, attitudes, and interpersonal communication skills are tested [22]. The validity and reliability of the OSCE have made this test the priority standard for evaluating practical clinical competence at a global level [23]. The experience of the use of the OSCE in the nursing discipline is perceived positively, and it is considered an objective evaluation test [24]. However, it has some limitations, such as evaluating teamwork, an aspect that is so important in professional practice today [25].

A recent integrative review of the literature with 24 articles concluded that gamification in nursing education positively affects students’ knowledge by motivating students and encouraging increased participation and involvement in classes, as well as greater student satisfaction; however, the review was not able to determine the degree of knowledge retention that occurs with this. Therefore, more studies are needed in this regard [26]. As other authors also conclude [12], there is a need to continue creating innovative teaching strategies and strategies based on simulation experiences [27]. No studies have been identified in the literature that carried out educational or evaluation strategies based on gamification through an escape room with simulation scenarios in the field of sexual and reproductive health-care nursing, and neither have experiences been identified in the literature that combine the OSCE and the escape room. Both these techniques can complement each other to arrive at a more adequate, objective, and comprehensive evaluation of all the aspects intended to be achieved in the nursing teaching process. For all these reasons, it was proposed to carry out a pilot study in which an OSCE would be conducted through gamification using simulators in teaching laboratories, evaluating the knowledge, abilities, attitudes, and skills that should be acquired in nursing sexual and reproductive health care. This study aimed to determine the usefulness of combining both techniques (OSCE and escape room) for evaluating this subject, as well as to understand the students’ assessment of this experience.

## 2. Methods

### 2.1. Design and Subject Selection

A cross-sectional observational study was conducted from November 2021 to December 2021 with 124 nursing undergraduate students from the University of Jaen who were enrolled in the sexual and reproductive health-care nursing course. There were no exclusion criteria.

To estimate the sample size, the population enrolled in the subject (N = 136) was considered the reference population. As a multiple-choice questionnaire was used, a prevalence of 50% was used as the most demanding criterion, a confidence level of 95%, and a precision or absolute error of 3%, resulting in a minimum sample size of 121 study subjects.

### 2.2. Development of the Escape Room

The students were randomly divided into groups of five. The assignment to groups was carried out through a computer algorithm. In the teaching laboratory, seven different simulators used for practical teaching seminars in sexual and reproductive health care were set up as stations in which a clinical case was developed that they had to solve. The following were the stations in which students had to show their knowledge, aptitudes, and skills.

- Station 1: Perform a gynecological examination and take a cervical smear.
- Station 2: Perform an obstetric examination during which the fetal heart rate could be auscultated, measure fundal height, perform the Leopold maneuver, estimate the likely due date and gestational age, etc.
- Station 3: Perform a breast examination, during which various abnormalities could be detected via inspection and palpation.
- Station 4: Male pelvic examination (prostate and testicular) so that the students could palpate, inspect, and conduct tests to detect benign prostatic hyperplasia, hydrocoele, tumors, etc.
- Station 5: Family planning methods consultation. Given the woman’s characteristics, medical history, and wishes, students need to assess the most appropriate contraceptive method for her.
- Station 6: Childbirth. The student has to identify whether the woman is in labor via vaginal exploration, fetal position, etc., and perform the basic procedures for assisting with childbirth.
- Station 7: Postpartum and puerperium complications. The students have to demonstrate their knowledge and skills regarding perineal injuries associated with childbirth, management of possible postpartum hemorrhage, etc.

Each group only used four stations. The maximum time to find the box with the room key, thereby solving the escape room, was 20 min. This box had a code of four numbers; each number had to be identified earlier in each station based on the examination, diagnosis, or care that the patient required at each moment according to the corresponding clinical case. A small clinical case was presented in each station, which could be for advice on contraceptive methods, cervical exploration, determining fetal station, etc., which would lead the students to act as if they were in a real situation using the simulators in question. This would help them to obtain the number that would later be used in the code of the box that contained the key. For example, in one of the clinical cases that were used as a clue, a situation was raised in which the woman had painful and regular uterine contractions for 2 h that did not settle or decrease with ambulation or immersion in warm water, and therefore came in for a consultation. To solve this, the students had to go to the station that simulated an obstetric emergency consultation and delivery room with the simulator and corresponding material and examine the woman to determine cervical dilation and the Hodge plane in which the fetus was located. The sum of both numbers was one of the code numbers that would help them open the box. Each clinical case was presented sequentially, and until the case in which they were busy was resolved (whether correctly or not), they were not presented with the next clinical case from another station. At no time were they told which of the seven stations the case corresponded to; they had to identify, depending on the characteristics of the case, which of the stations they should go to carry out the procedures, examinations, etc., that would help them to determine the code number of the box. In order to escape, they had to have correctly identified the numbers and therefore had identified the problem appropriately and correctly performed the procedure, the technique, and the appropriate care in each case. If there was an error at any point, that is, the problem had not been identified, or the proper care, procedures, or techniques had not been carried out, the box did not open, and therefore it was not possible to escape. The evaluation was carried out through two criteria: whether they had escaped or not (satisfactorily resolved the clinical cases) and the time spent. A higher rating was assigned to those who spent less time solving the escape room. In addition, while students carried out the techniques, procedures, and care, the teacher observed and used a checklist to verify the correct performance of the techniques, procedures, and care. If this had not been done correctly, the group had a 2-second penalty for each item on the checklist that they had not fulfilled.

### 2.3. Information Sources

As a source of information, a self-prepared questionnaire was used that included 48 items (1 open question, 47 closed questions) on sociodemographic and academic characteristics, as well as questions related to the escape room experience and the GAMEX (Gameful Experience in Gamification) scale, which measures the gamified experience. This scale is made up of 27 questions with Likert-type response options (1 = “totally disagree” and 5 = “totally agree”), with a maximum of 30 points. It consists of six main dimensions (maximum 5 points per dimension): enjoyment/fun, which measures the degree of user enjoyment with the gamification experience (6 items); absorption, which indicates the degree of engagement in the experience and evasion of the surrounding environment, in addition to the awareness or not of the notion of time while the experience lasts (6 items); creative thinking, which analyzes the degree of imagination or creativity that the user perceives that they develop during the experience (4 items); activation, which measures the degree of activity that the user considers they have developed during the experience (4 items); absence of negative affect, which measures whether users have expressed or felt negative emotions while playing, such as frustration (3 items); and finally dominance, which considers the feeling of being in control and analyzes the dominance or confidence that the user has in themselves during the experience (4 items). There was a maximum of 5 points per item. GAMEX has demonstrated its reliability as an instrument for collecting information on gamified experiences [28], with Cronbach’s alpha values greater than 0.90 globally and for each of the instrument’s dimensions.

The questionnaire had been piloted previously. The piloting was carried out by selecting a group of 10 students. This sample was heterogeneous in terms of age, sex, and previous experiences in clinical practices and gamification. It was administered within 24 h of carrying out the escape room test. Before starting the questionnaire, the students had to read an information sheet about the study and objectives and were able to voluntarily provide their consent to participate in the study. Once they agreed to participate, they were given the instructions for completing the questionnaire.

The following variables were collected.

The independent variables were: age, gender (male, female, non-binary), academic record (average grade obtained so far in the degree), access degree (entrance exam, test for people over 40 years of age, vocational training degree), and experience with an escape room (yes, no). The dependent variables were the GAMEX scale with its dimensions and various questions about the experience with the escape room and its academic usefulness.

### 2.4. Statistical Analysis

First, a descriptive analysis was performed using absolute and relative frequencies for categorical variables and mean with standard deviation for numerical variables. Next, a bivariate analysis was carried out using the Student–Fisher *t*-test to relate the GAMEX scale scores to various sociodemographic and academic variables. Mean differences (MDs) were estimated with their respective 95% confidence intervals (95% CIs).

### 2.5. Legal and Ethical Considerations

This project received the approval of the ethics committee of the University of Jaen (DIC.21/7.PRY). All students included in the study signed an informed consent.

All data were treated confidentially in accordance with Organic Law 3/2018 of December 5 on the Protection of Personal Data and Guarantee of Digital Rights, keeping its strict confidentiality and its non-access to unauthorized third parties and Regulation (EU) 2016/679 of the European Parliament and of the Council of 27 April 2016 on Data Protection (GDPR).

## 3. Results

Of the total number of students enrolled in the course (N = 136), 124 participated (91.1%). Most (80.6%, 100) were women with a mean age of 21.7 years (SD = 6.77 years). The majority (88.7%, 110) had not previously had clinical rotations related to the subject, and 33.9% (42) had previously participated in an escape room outside the university (Table 1).

**Figure 1:**
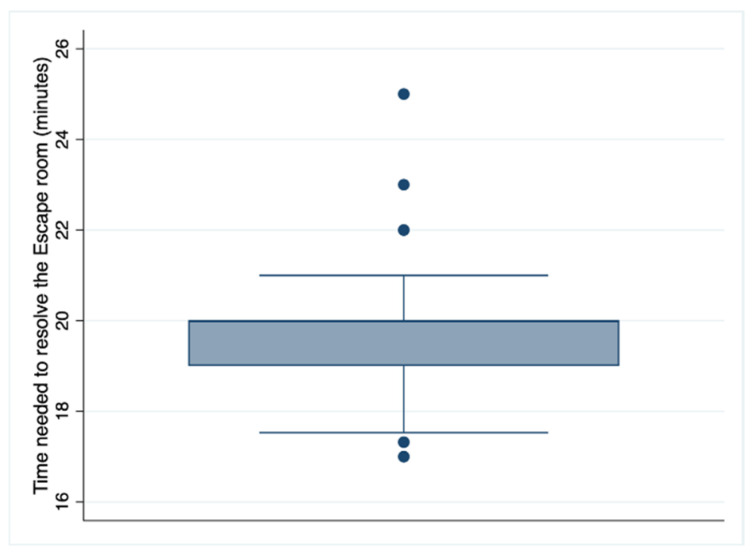
Distribution of the time needed by students to solve the escape room.

Table 2 shows that as far as the experience and perception of the usefulness of the escape room are concerned, 100% of the students considered it an adequate methodology in this course and an innovative teaching methodological tool. Most (86.3%, 107) completely agreed that participating in the exercise helped them learn about the subject, and 80.6% (100) completely agreed that it would help them retain knowledge for the final exam. In this sense, 80.6% (100) of the students completely agreed that they remembered and applied the knowledge of the subject during the game, and 91.9% (114) completely agreed that there should be more gamification experiences in nursing education. Regarding the escape room as a motivational tool, a total of 119 students (96%) agreed and completely agreed, and all (124) of the students felt satisfied or very satisfied with the experience. Curiously, although 36.3% (45) could not solve the escape room (the evaluation), 98.4% (122) would recommend this experience to their friends and colleagues. Figure 1 shows the distribution of the time used by the students to solve the escape room.

**Figure 2:**
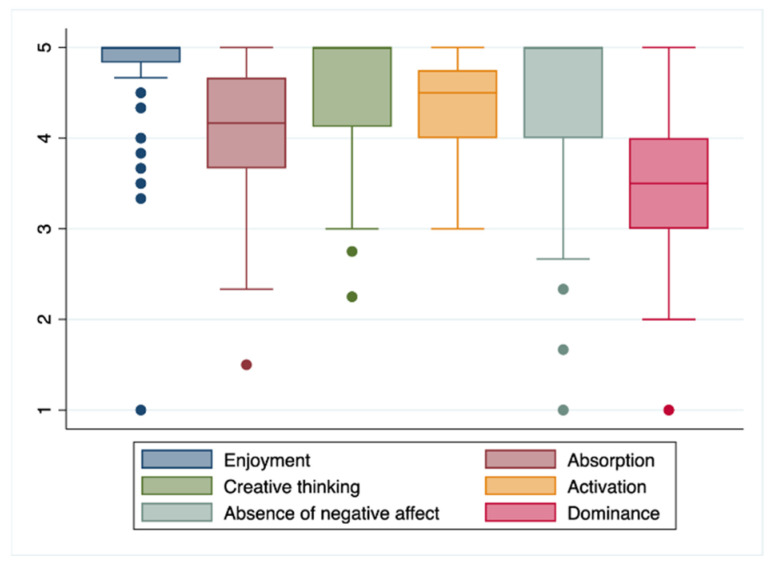
Distribution of the scores on the different dimensions of the GAMEX scale.

In Table 3, the gamified experience is assessed with the GAMEX tool, observing a total average score of 25.6 points (2.36) (maximum 30 points). The dimension with the best evaluation was “Fun”, with an average score of 4.7 points (0.49), followed by “Critical thinking”, with 4.2 (0.59); the distribution of the score in the different dimensions of the GAMEX scale can be seen in Figure 2. The dimension with the worst evaluation was “Domain”, with 3.5 points (0.80) (Table 3).

Finally, the relationship between numerous factors and the scores of the gamification experience was evaluated. Women presented a statistically better score than men (mean difference: 1.58; 95% CI: 0.55, 2.61), as well as those who had made previous clinical rotations related to the subject (mean difference: 1.22; 95% CI: 0.53, 2.38) (Table 4).

## 4. Discussion

The current study was conducted with the goal of assessing the effectiveness of integrating teaching methods, notably objective structured clinical examination (OSCE) and escape room strategies, within a simulation-based environment for the instruction of sexual and reproductive health care. A notable outcome of this integration was the high success rate among participants in the assessment, alongside unanimous acknowledgment by students of the gamified OSCE as an innovative and fitting approach within a simulated setting. The majority of students concurred that this method not only facilitated experiential learning but also significantly aided in memory retention and knowledge recall pertinent to the subject’s final examination. Moreover, the students overwhelmingly recommended this educational experience to peers, attributing their satisfaction to its role as an engaging tool that fostered interest in the subject matter, thereby encouraging further study. Within the GAMEX scale evaluations, the aspects of enjoyment and critical thinking received the highest ratings. Interestingly, the gamification experience garnered more favorable evaluations from female students and those who had previously completed clinical rotations in fields related to sexual and reproductive health, such as obstetrics and gynecology.

The percentage of students who passed the practical part of the subject through this experience was similar to the previous year using the method of a multiple-choice test. This supports the usefulness of the OSCE test using an escape room to evaluate the subject. Along these lines, Roman et al. [28], in a study on 95 nursing students in their final year, found the escape room useful as an evaluation tool, emphasizing that this method develops teamwork and communication [29]. Other authors, such as Larcom et al. [30], also considered the escape room useful as an evaluation instrument. If we also consider the findings from authors such as Roma et al. [31] that report OSCE through games as an adequate evaluation method, we can deduce that our results of a combination teaching tool (OSCE + escape room) in a simulation environment are very much in line with the results of these authors who have worked on these methodologies independently [30,31].

In addition to being an evaluation method that has demonstrated its usefulness, it should be noted that even the students themselves considered it appropriate. This may be because they recognized that with this methodology, they can learn and knowledge is retained, facilitating the consolidation and application of knowledge, as other authors have also identified [21,32,33]. However, in an integrative review of the literature including 24 articles, it was concluded that despite the positive effect that gamification has on knowledge, it was not possible to determine the degree of retention of knowledge that students had with the use of gamification [26], though this review includes a large number of gamification techniques and not only the escape room.

In line with what was observed by other authors, there is a demand among students to implement gamification strategies such as the escape room in nursing education. In addition, students responded with favorable comments regarding the experience, coinciding with other authors [18]. Students even considered it innovative and motivating to continue learning and studying and would recommend it to third parties. Overall, their assessment regarding the experience was very satisfactory, similar to that already reported by other authors [19].

In the assessment using the GAMEX scale, this methodology highlighted both critical thinking and the fun dimension, in line with that found by several other authors [19,34,35,36]. Parker reported that escape rooms are a great opportunity to generate critical thinking in students [36], and Molina-Torres et al. [18] found that the “enjoy playing” of the “fun” dimensions received the highest score in the study carried out in Spain with 248 first-year nursing degree students [19]. Regarding the mean score of each of the dimensions of the GAMEX scale, all had a mean score higher than four points, one point higher than what Anguas-Gracia et al. found in an escape room experience on community health in nursing students [21].

Interestingly, women obtained a higher score in our gamification experience, as did those students who had already rotated through obstetrics and/or gynecology services (services related to the content of the sexual and reproductive health-care nursing subject). We have not found results in the literature studying this gender association, although other authors did identify gender differences in the domain dimension, but not in the total score, as mentioned above [21]. The experience gained from having rotated through those where the care, procedures, and techniques developed in the subject are carried out may have been the reason for this association. The average grade, not solving the escape room, or having previous extra-academic experience in an escape room were not found to influence the score of the gamification experience.

Among the study’s strengths was the participation of almost the entire student population: only 12 students declined to participate for different reasons (isolation due to COVID-19, family reasons, etc.). This means that a possible selection bias associated with non-response cannot have had a decisive influence on the results. In the same way, there are no indicators that suggest that the students who did not respond would have responded differently from those who did. A memory bias can be ruled out due to the brief time elapsed between the escape room experience and information collection (less than 24 h). The questionnaire used had been previously piloted, and the GAMEX scale is validated [28] and has already been used in a population similar to ours [19,21].

The results obtained support the implementation of this methodology as the most appropriate for evaluating the subject. Nonetheless, we propose future lines of research to explore whether this new form of evaluation influences the development of clinical practices that students will develop in health centers where sexual and reproductive health care is provided.

## 5. Conclusions

In conclusion, integrating objective structured clinical examinations (OSCEs) with gamification techniques, such as escape rooms incorporating scenario simulations, proves to be an effective method for assessing the practical components of the sexual and reproductive health-care nursing course. Students found this approach both appropriate and innovative, giving it high marks for its educational value. They recommended this methodology enthusiastically, expressing satisfaction with how it facilitated learning, knowledge retention, and application. Furthermore, it served as a significant motivational tool, encouraging continued study in the field. All dimensions evaluated by the GAMEX scale received exceptional scores, underscoring the comprehensive benefits of this educational strategy in enhancing student engagement and learning outcomes.

## Figures and Tables

**Table 1:** Sociodemographic and academic characteristics of the students.

Variable	n (%)	Mean (SD)
**Age**		21.73 (6.77)
**Sex**		
Male	24 (19.4)	
Female	100 (80.6)	
**Mean grade**		7.94 (0.59)
**Highest academic year enrolled**	109 (33.5)	
Second	118 (96.0)	
Third	1 (0.8)	
Fourth	4 (3.2)	
**Other qualifications related to health sciences**		
No	92 (74.2)	
Technical institute training in nursing care	2 (1.6)	
Advanced technical training	26 (21.0)	
Other degree	4 (3.2)	
**First time enrolled in this subject**		
No	2 (1.6)	
Yes	122 (98.4)	
**Previously completed a clinical rotation related to this subject**		
No	110 (88.7)	
Yes	13 (10.5)	
**Participated in an escape room outside the university**		
No	82 (66.1)	
Yes	42 (33.9)	
**Experience with gamification in other subjects**		
No	26 (21.0)	
Yes, but external to the university	95 (76.6)	
Yes, in the university	3 (2.4)	

**Table 2:** Experience and perception of the usefulness of the escape room for the subject.

Variable	n (%)
**Do you consider the escape room game to be an appropriate learning method for this course?**	
No	0 (0.0)
Yes	124 (100)
**Do you consider that the escape room is an innovative teaching tool?**	
No	0 (0.0)
Yes	124 (100)
**Playing helped me learn about the subject**	
Completely disagree	0 (0.0)
Disagree	1 (0.8)
Neither agree nor disagree	1 (0.8)
Agree	15 (12.1)
Completely agree	107 (86.3)
**I think the game will help me in the exam**	
Completely disagree	0 (0.0)
Disagree	1 (0.8)
Neither agree nor disagree	3 (2.4)
Agree	20 (16.1)
Completely agree	100 (80.6)
**I remembered and applied my knowledge of the subject during the game**	
Completely disagree	0 (0.0)
Disagree	0 (0.0)
Neither agree nor disagree	0 (0.0)
Agree	14 (11.3)
Completely agree	100 (80.6)
**There should be more games like this during the nursing degree**	
Completely disagree	0 (0.0)
Disagree	0 (0.0)
Neither agree nor disagree	1 (0.8)
Agree	9 (7.3)
Completely agree	114 (91.9)
**The game motivated me to continue studying, even though the exam is several weeks away**	
Completely disagree	0 (0.0)
Disagree	1 (0.8)
Neither agree nor disagree	4 (3.2)
Agree	24 (19.4)
Completely agree	95 (76.6)
**Overall, how satisfied were you with the escape room experience in this subject?**	
Not at all satisfied	0 (0.0)
Not satisfied	0 (0.0)
Somewhat satisfied	0 (0.0)
Satisfied	9 (7.3)
Very satisfied	114 (91.9)
**Did you manage to solve the escape room?**	
No	45 (36.3)
Yes	79 (63.7)
**How much time did you need to solve it? Mean minutes (SD)**	19.6 (1.36)
**Would you recommend it to a friend and/or classmate?**	
No	1 (0.8)
Yes	122 (98.4)
Maybe	1 (0.8)

**Table 3:** Evaluation of the gamification experience using the GAMEX tool.

Dimensions and Items	Mean (SD)
**Enjoyment**	4.7 (0.49)
En1—Playing was fun	4.9 (0.45)
En2—I liked playing	4.9 (0.45)
En3—I really enjoyed playing	4.8 (0.61)
En4—My experience with the game was pleasant	4.7 (0.64)
En5—I think the game is very entertaining	4.9 (0.46)
En6—I would play this game of my own accord, not just when asked	4.5 (0.83)
**Absorption**	4.1 (0.72)
Ab1—Playing made me forget where I was	4.1 (0.99)
Ab2—I forgot where I was as soon as I started playing	4.2 (0.94)
Ab3—After playing, I felt like I was returning to the “real world” after a journey	3.8 (1.00)
Ab4—Playing “took me away from it all”	4.0 (0.93)
Ab5—While playing, I was completely oblivious to everything around me	4.0 (0.89)
Ab6—While playing, I lost track of time	4.6 (0.63)
**Creative thinking**	4.5 (0.59)
Ct1—Playing captured my imagination	4.6 (0.70)
Ct2—While playing, I felt creative	4.5 (0.71)
Ct3—While playing, I felt like I could explore things	4.6 (0.53)
Ct4—While playing, I felt adventurous	4.4 (0.82)
**Activity**	4.3 (0.54)
Act1—While playing, I felt active	4.4 (0.82)
Act2—While playing, I felt nervous	4.8 (0.43)
Act3—While playing, I felt frantic	4.0 (1.12)
Act4—While playing, I felt excited	4.5 (0.62)
**Absence of negative affect**	4.4 (0.87)
Ana1—While playing, I felt annoyed	4.5 (0.92)
Ana2—While playing, I felt hostile	4.5 (1.01)
Ana3—While playing, I felt frustrated	4.2 (1.06)
**Dominance**	3.5 (0.80)
Dom1—While playing, I felt dominant/I had the feeling I was in charge	2.8 (1.16)
Dom2—While playing, I felt influential	3.7 (0.99)
Dom3—While playing, I felt independent	3.5 (1.17)
Dom4—While playing, I felt confident	3.9 (0.87)
**Total score**	25.6 (2.36)

**Table 4:** Relationship between diverse factors and the gamification experience evaluated by GAMEX.

Variable	Mean (SD)	Mean Difference 95% CI	*p*-Value
**Sex**		1.58 (0.55, 2.61)	0.003
Male	24. 4 (2.74)		
Female	25.9 (2.15)		
**Previously completed a clinical rotation related to this subject**		1.22 (0.53, 2.38)	0.041
No	25.5 (2.40)		
Yes	26.7 (1.81)		
**Mean grade**		0.11 (−0.96, 0.75)	0.803
<8	25.7 (2.49)		
≥8	25.6 (2.28)		
**Participated in an escape room outside the university**		0.07 (−0.81, 0.96)	0.870
No	25.6 (2.36)		
Yes	25.7 (2.37)		
**Did you manage to solve the escape room?**		0.25 (−0.62, 1.12)	0.571
No	25.5 (2.45)		
Yes	25.7 (2.31)		

## Data Availability

Data will only be made available via a request to the authors.

## Public Involvement Statement

The participants in the study were nursing students at the University. Consent was obtained from all participants to use the data obtained in the research to prepare an article and its subsequent publication.

## Guidelines and Standards Statement

This manuscript was drafted against the STROBE for observational studies research.
